# Dimensions of challenges in transformation from traditional to integrated modular curriculum - Experiences from Pakistan

**DOI:** 10.12669/pjms.39.6.6730

**Published:** 2023

**Authors:** Sajid Aziz, Gohar Wajid, Rehan Ahmed Khan, Fatima Zia Zaidi

**Affiliations:** 1 Sajid Aziz, MBBS, FCPS, MME Nawaz Sharif Medical College, Gujrat, Pakistan; 2 Gohar Wajid, PhD. Consultant, Health Professions Education, World Health Organization, Cairo, Egypt; 3 Rehan Ahmed Khan, FCPS, FRCS, MHPE, PhD. Riphah International University, Rawalpindi, Pakistan; 4 Fatima Zia Zaidi, MBBS, MPhil, MME University College of Medicine, The University of Lahore, Lahore, Pakistan

**Keywords:** Curriculum Transformation, Integrated Curriculum, Curriculum Implementation, Challenges, Change Management

## Abstract

**Objective:**

The scarcity of successful implementation of integrated modular curriculum in Pakistan created a lack of evidence-based insight into the process of curriculum transformation. We explored the issue by exploring challenges faced by faculty in implementing integrated modular curriculum.

**Methods:**

An exploratory qualitative study was conducted in 2019-2020, data collected from November 2019 to August 2020, at undergraduate medical colleges in Lahore and Rawalpindi, with semi-structured online interviews of well experienced 11 faculty members. Two levels of faculty were sampled; one completed a successful implementation in two medical colleges, and another in transition process. Transcribed interviews were analyzed on Atlas.ti software.

**Results:**

Challenges were identified in five inter-connected dimensions: integrated modular curriculum (IMC) development, implementation, faculty transformation/change, institutional and regulatory body context.

**Conclusion:**

The curricular shift precipitates demoralizing challenges at multiple levels & directions. The identified themes and connections provide the framework for a well-informed curricular shift.

## INTRODUCTION

In Pakistan, most medical institutions use a traditional subject-based curriculum, with a movement towards adopting integrated modular curricula. Overburdened with information, the students tend to forget the already learned material as they progress from the basic sciences to clinical application and integrative learning solely relies on individual student’s abilities. This fragmented learning and ungauged integration can subsequently have implications on patient care.^1^

Unfortunately, transformation towards integrated curriculum is full of off-putting challenges. These arise from accreditation constraints, unfitting curricular framework and design, faculty capacity and resistance, student’s response to change, management issues and deficient resources, both human and material.^2^-^6^ Other constraints may include motivation and perseverance that are needed on part of stakeholders for success and sustenance.^7^,^8^

Hence, an institution must have a clear vision to make a move towards integrated curriculum. The exponential increase of medical colleges in Pakistan and the dearth of experienced medical educationists can stall the process of change at crossroads.^9^ The objective of this study was to identify the challenges experienced by faculty in transformation from subject based curricula into integrated modular curricula in Pakistani medical colleges.

## METHODS

An exploratory qualitative study was conducted during 2019-2020, for detailed analysis of experiences and challenges faced by the faculty of two private undergraduate medical colleges that had transformed discipline-based curriculum into integrated modular curriculum (from Lahore and Rawalpindi) and one government college still in transition (from Peshawar). Duration of study was 13 months from institutional review board approval (Ref: ERC/02/19/07) to manuscript writing.

The interview guide was developed after a systematic literature review of last ten years using PRISMA flow chart. The problems identified in the selected studies led to the development of themes for our guide. These themes included understanding of integrated curriculum, implementation & monitoring of integrated curriculum, and transformational change management. The interview guide was refined through expert validation and pilot testing.

Ensuring confidentiality and informed consent, online interviews were recorded through “ZOOM” platform. After verbatim transcription of the interviews, the software Atlas.ti version 7.5.7 was used for thematic analysis based objectively on participant’s narratives only, which was read and re-read before coding. Respondent validation and data triangulation was carried out through involving respondents. Two private medical colleges in different cities that had, at different points of time, completed curriculum transformation, and an in-transition government medical college were sampled. Moreover, perspectives from different levels of change leaders & implementers among faculty were obtained i.e., heads of departments, medical educationists, module directors, clinical/basic faculty and junior/senior faculty.

## RESULTS

The faculty characteristics are given in Table-I emphasizing variability & depth of perspectives. The thematic analysis resulted in a total of 235 codes. Initially 306 and 171 codes developed from faculty interviews in Lahore & Rawalpindi respectively and 212 codes from faculty interviews in Peshawar. In second cycle these were reduced to total 461 codes. After further deliberations on linkages and overlap reduction total 235 codes were finalized. The codes having similar basis were grouped together into related entities like small group teaching, infrastructure issues, faculty resistance, assessment challenges, change management, disciplines limitations, Department of Medical Education, faculty development, faculty work, implementation autonomy, interdisciplinary communication, integrated modular curriculum issues, implementation planning, implementation receipt, leadership attributes, module guides and learning outcome issues, module management, need for integration, PM&DC issues, qualifications for reform implementation, student attributes, teacher attributes, teacher student relationship etc.

Table-II shows 32 different categories identified from these codes, which are segregated into 10 sub-themes. Eventually five themes emerged revealing different dimensions of faculty challenges in the integrated modular curriculum (IMC) transformation i.e., challenges of IMC development, challenges of IMC implementation, challenges of transformation & change, challenges in institutional context and challenges in the context of national regulatory body. Table-III shows a few representative quotations out of total 968 quotations.

**Table-I:** Participants Characteristics

Participants Characteristics		n = 11	Percentage
Age group	< 40 years	06	55%
	> 40 years	5	45%

Academic qualification	Bachelor’s degree (medical)	11	100%
	Specialization (subject)	10	91%

Medical education qualification (enrolled / completed)	Certificate	02	18%
	Masters / Diploma	10	81%
	PhD / PhD scholar /Post doc	04	36%

Curriculum transformation experiences	Curriculum implementation	11	100%
	Curriculum development	06	55%
	Change leadership role	05	45%

Roles in modular curriculum	Facilitators	05	45%
	Module Coordinators	03	27%
	Module Directors	05	45%
	Committee members	06	55%

Roles in traditional curriculum	Demonstrator	01	9%
	Assistant professor	04	36%
	Associate professor	01	9%
	Professor	04	36%

Experience in traditional teaching	> 5 years	08	73%
	< 5 years	03	27%

Experience in integrated modular Teaching	> 5 years	05	45%
	< 5 years	06	55%

Discipline	Basic	08	73%
	Clinical	03	27%

DME* experience	Full time	05	45%
	Assignments / part time	06	55%

Current institution background	Government sector	03	27%
	Private sector	08	73%

* Department of Medical Education.

**Table-II:** Organization of Categories, Subthemes and Themes.

Categories	Subthemes	Themes
IMC development need IMC development outcome goals IMC development foresight IMC development fault lines	Rational discourse for IMC development	Challenges of IMC development

Interdepartmental collaboration recognition Interdepartmental collaboration impediments	Collaborative teamwork task in IMC development	

The content issues of module development Integrated assessments issues Subject limitations in integrated modules	Bottlenecks of IMC development	

Faculty development outcome goals Faculty development impediments Teacher & student adaptations Faculty workload analysis	Challenges of aligning implementers to integration level	Challenges of IMC implementation

Implementation essential alignments Implementation operations management Implementation preparedness checklist Robust DME with clear mandate Module management incongruence issues Module management integration tasks Small group teaching needs	Challenges of aligning structures to integrated design	

Implementation receipt antecedent tasks Implementation deferred verification Implementation real-time verification	Challenges of establishing implementation receipt system	

Change strategy Change governance Change leadership	Change process oversight task	Challenges of transformation and change

Change support needs Change resistance appreciation	Change process facilitation task	

Institutional attributes Institutional setbacks	Need for institutional conformity	Challenges in institutional context

PM&DC issues Curriculum specific issues	Need for holistic guidance & monitoring systems	Challenges in context of the national regulatory body (PM&DC)

*Integrated Modular Curriculum.

**Table-III:** Representative quotations

Theme	Subtheme	Representative codes & quotations
Challenges of IMC development	Rational discourse for IMC development	Traditional teaching inadequacy “It was a rotten system in which all the struggle was done by the students. You listen to a lecture or don’t listen, with few exceptions, it was of no significance. Also the assessment system was by chance. In long cases they may ask two questions and give you full marks or ask twenty it was all your luck.” excerpt P 2: 2:110 , (144:144) Facilitate integrative learning “… I see that they are more facilitated and can easily study a topic as they get information from all perspectives that were not the case with traditional system where the information was scattered in different subjects and at different times … It is easy for them to grasp if they want to and I again repeat if they want to!” excerpt P 1: 1:82 , (122:122)

	Collaborative teamwork task in IMC development	Breaking silo effect “Every faculty member was looking into their individual part in curriculum. Now they are engaged and planning for the whole curriculum. ” excerpt P 3: 3:68 , (56:56)

	Bottlenecks of IMC development	Need for alignment to integration outcomes “If assessment is not aligned to integration then the quality of teaching is also going to suffer… Unless we (assess) examine the module in relation to integration, the pure and true integration is not possible.” excerpt P 1: 1:302 , (423:423)

Challenges of IMC implementation	Challenges of aligning implementers to integration level	Capacity building aligned to needs “…We have to know the specific methodology we are using in a particular method for example skill lab should be hands on, SGD should not be in the form of interactive lecture and so on…The teacher must adapt his style and approach according to the methodology or the specific method.” excerpt P 1: 1:138 , (205:205)

	Challenges of aligning structures to integrated design	Establish implementation feasibility “Yes, definitely, integrated curriculum needs different infrastructure, small group rooms, more faculty and more resources than traditional system.” excerpt P 1: 1:304 , (425:425)

	Challenges of establishing implementation receipt system	Capacity building for monitoring “The point is valid but there is no capacity building for this job (monitoring of implementation). We should have this like in CIPP model. However we have done our context analysis to know our strengths and we are developing inputs based on this.” excerpt P 3: 3:224 , (112:113)

Challenges of transformation and change	Change process oversight task	Establish feedback loops “We developed five committees to maintain a feedback loop during change process. These include curriculum, assessment, program evaluation, faculty development and research committees.” excerpt P 2: 2:14 , (10:10)

	Change process facilitation task	Neophobia & insecurity “Then there are insecurities, effort needed for learning new things, fear of failure to learn and the fear that juniors may become more competent in new system.” excerpt P 2: 2:160 , (210:210) Ensure autonomy & inclusiveness “But it was good enough to give it a try to develop their trust on us by giving them autonomy, giving the ownership and engaging them and inclusiveness. This was quite challenging because it was quite time consuming activity for me and I also faced backfire and criticism and also lots of fears and I had to address them all.” excerpt P 3: 3:57 ., (48:48)

Challenges in institutional context	Need for institutional conformity	Investment on faculty development “Millions of rupees were spent on faculty development, ICME conference, our visits, our training, even on our PhD.” excerpt P 2: 2:170 , (222:222) Red tapism “As I told you and you may know how the bureaucracy and red-tapism works and despite the efforts for the changes you want to bring in, it is not possible to improve in the government setup.” excerpt P 3: 3:26 , (15:15)

Challenges in PM&DC context	Need for holistic guidance & monitoring systems	Political instability issues “As you know, it is an unstable body, sometimes it is PMC and then it is again PM&DC.” excerpt P 2: 2:64 , (75:75) Lack of holistic inspection “PM&DC should check the infrastructure and faculty number but more importantly should see that the program is implemented according to the soul and spirit of their written curriculum.” excerpt P 2: 2:174 , (225:225)

## DISCUSSION

The question of challenges experienced by the faculty in traditional curriculum transformation to IMC is answered holistically. The challenges are exclusive to the faculty of target colleges and transferability is limited, however, are comparable with the challenges mentioned in related local and international studies.^5^,^6^,^10^-^13^ The local studies provided perceptions on the design and implementation difficulties but lacked in depth analysis. The international studies mentioned frameworks from the stand point of change management models, strategic planning, standards and inhibitors and implementation models, emphasizing preemptive awareness of challenges and an organized holistic approach but are difficult to comprehend by those without medical education background.

**Fig.1:**
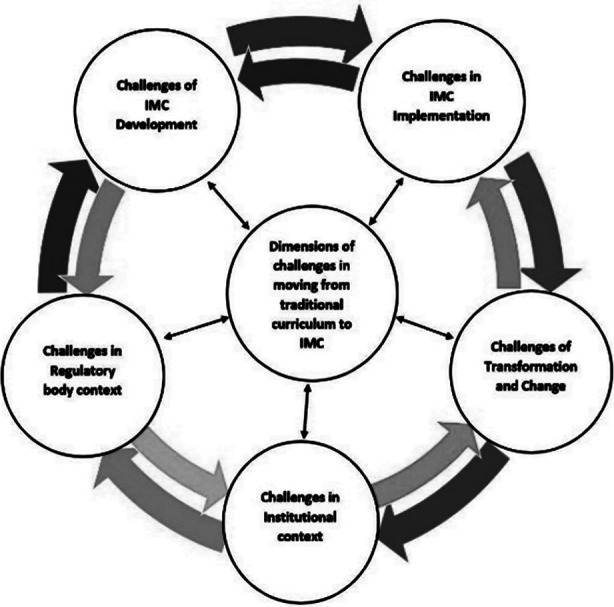
Interconnected dimensions of challenges.

Our study explored challenges at macro and micro levels principally in five main dimensions i.e., integration, implementation, change, institution and regulatory body (PM&DC). It further added that these challenges are rather dynamically interconnected and changes in one dimension create challenges in other dimensions too. The challenges must be addressed in an all-inclusive manner (Fig.1) for tackling uncertain complexities; hence both forward planning and backward planning is needed.^14^ To facilitate comprehension and alignment the results are interpreted under four well known core elements of the curriculum i.e., aims or objectives, content or subject matter, methods or procedures and evaluation or assessment.

In the development of IMC, the aim challenged nothing else but a focused rational discourse on traditional teaching inadequacy and informed decision on the development of integrative learning models. Primary challenges included discarding subject lines and shutting off avenues of rote learning. High quality integrated module and effective small group teaching became pivotal targets. After having IMC in hand, it is imperative to develop a clear road map for implementation. First milestone, the challenge of aligning the implementers to integration level that burdened faculty with extra workload, mainly due to double curriculum in transition, small groups, multitasking, work-fatigue, frequent assessments and early clinical engagements with hectic patient load. Second milestone**,** the challenge of adaptation to new curricular philosophy with change of traditional mindset and settlement for new roles; especially to represent subject in relation to overall module.^7^ Third milestone, challenge to institute faculty development program with clear outcomes of capacity building aligned to needs, capacity for collaborative teamwork, cohort of experts for sustainability, module development task, neophobia prevention, sensitization to recent trends and unlearning traditional teaching practices. The process was not without impediments like focus on practicable content only, participation hurdles, and perpetual need for training, time, effort, and money.^5^,^6^,^15^ The fourth milestone, challenge of deciding on financial non-feasibility, degree of fidelity of implementation and alignment to regulatory body criteria. Curricular change aim would have been a preplanned change model but the participants never experienced an informed change intervention. However, the challenges they faced can be organized as objectives of change i.e., sensitization, process initiation, consolidation and refinement, process conclusion, internalization and a perpetual cycle of change. The organizational setup was challenged to aim for conformity to the IMC design and PM&DC regulations, which in turn to facilitate it.

IMC content development challenge was elimination of bottlenecks by defining criteria for content inclusion, exclusion, sequencing, validity, overload/renewal, learning objectives (LOs) overlapping, LOs restricted teaching and subject primacy issues. This summoned a need for expert validated content, integrated within and across different modules and phases. The implementation content encompassed physical/organizational implementation structures needed for the roadmap like implementation hierarchy, faculty job description to avoid overlapping responsibilities, subject’s faculty strength to module needs, trained faculty hiring, fully utilizing existing faculty, skill labs resources, flexible use of physical space and a systematic ward teaching including fixed students to ward ratio. The clinical subjects remained a challenge to plan implement structures for want of necessary departmentalized existence. Structures challenges also involved module authority, holistic accountability, module-based identity, structured linking of topics and task-specific training. The faculty resistance is the core content of transformational challenges that manifested as willful lacking implementation, hostile rigidity, and even resignations. The factors that lead to this resistance included non-inclusive decision making, rigid readymade curriculum, culture of silos, changing routine, avoidance of extra work, compensation, private practice, telescopic perspectives, age factor, by chance teachers, power shift, neophobia and insecurity, stuck to status quo and conflicts with teaching content. The content challenges of organizational and regulatory body included red-tapism, lacking legal cover, inadequate ancillary facilities and lacking DME capacity.^13^,^15^

The systematic method of IMC development involved three challenges. Firstly, bid for extra effort/resources, valid literature guidance, inclusive development, multi-dimensions effect. Secondly confront fault lines by establishing curricular alignments to faculty’s implementation capacity, regulatory body obligations, a valid template like Harden’s integration ladder, timetable for topic sequencing and instructional strategy for holistic learning.^16^ Thirdly establish interdepartmental collaborative-teamwork as a linchpin to ensure modular function by breaking silos and appreciating team effort.^7^,^17^

The lack of mindset, role understanding, uniform medical education background, co-ordination and leadership considered as main impediments.^5^,^6^ The method for a planned IMC implementation needed operation management approach with staged implementation strategy. The operationalization needed secured validated course modules, consultant oversight, DME to ensure fidelity, curriculum self-alignment structures, sanctioned human resources, infrastructure, internet/IT support and statutory budget allocation.^3^,^13^

The DME acting as a nerve center, confronted with the need for a full-time faculty and a clear mandate with challenges to provide leadership, curriculum development, faculty collaboration, effective communication, faculty training, operationalize curriculum and enable small group teaching.^8^ The structured method for the transformational change challenged multi-tiered leadership and establishment of support structures. The specific challenging leadership attributes at the highest level were clear concept of process, people and purpose, firm belief in change, confidence of stakeholders, involvement in process, open to diverse views and adaptive capacity. At faculty level academic credibility, coaching skills, good interpersonal skills, good managerial skills and being a dedicated self-directed learner are challenges of leadership. The challenges of establishing support to change process included open communication, autonomy, inclusiveness, collaborative teamwork, ownership, open faculty discussions, incentives, cultural embedment, top-down diffusion of change and carrot & stick approach. The red flags included discard of fair criticism, patchy support and fixation with faculty resistance.^10^ The challenges of a planned institutional and regulatory body method asked for financial autonomy, commitment, appraisals, freedom to plan & implement, shared value system and investment on faculty development.

The challenges of evaluation and assessment of IMC development necessitated for piloting and a prior framework for curriculum impact on students. The integrative assessment demonstrated a choking point partly due to the need for a functional assessment cell but mainly owing to the grinding challenges of lacking alignment to integration holistic outcomes and lacking in-depth subject-based evaluation. The perpetual appraisals and selective student’s effort because of weighted based scoring remained unchanging challenges.^1^,^13^

The evaluation and assessment of implementation challenged for the implementation receipt system. The fundamental challenge was to acknowledge set-back of its absence. Evaluation needed the tasks of separate hierarchy, resource allocation, capacity building, valid & reliable feedback, recording recurring gaps and identifying quality constraints. The deferred verification included assessment results, evaluation outcomes and stakeholders’ feedback.^1^,^18^,^19^ The need for real-time monitoring surfaced by the need for immediate rectifications but was hindered by lacking feasibility and acceptability. The challenges of evaluation and assessment of transformational change included hiring consultant oversight, assigning leadership, establishing feedback loops for timely conflict resolution. The regulatory body (PM&DC) needed to provide oversight and evaluation for the institution and for the process. This remained an insurmountable challenge due to political instability, fitful progress, restricted framework for integration, lack of holistic inspection and rudimentary DME.^4^,^19^

### Limitations of the study

Methodological triangulation was not feasible due to COVID-19 lock down and sensitive nature of information. Further research is needed to explore the medical college’s strategic position in the existing overall healthcare system after this curriculum shift.

## CONCLUSION

The challenges are multi-dimensional and have multiple facets. This needs involvement of all stake holders at each level to communicate and inclusively work for the curriculum transformation because it is not a one man show. The identified inter-connected themes can guide a framework to employ all aspects of the curriculum transformation in a stepwise, guided, and meaningful manner.

### Authors’ Contributions:

**SA:** Conception, design, data collection, manuscript writing. Responsible for accuracy of work.

**GW, RAK:** Conception & design, manuscript review & approval.

**FZZ:** Data collection, manuscript writing.
